# Hormonal Receptor Status Determines Prognostic Significance of FGFR2 in Invasive Breast Carcinoma

**DOI:** 10.3390/cancers12092713

**Published:** 2020-09-22

**Authors:** Marcin Braun, Dominika Piasecka, Bartlomiej Tomasik, Kamil Mieczkowski, Konrad Stawiski, Aleksandra Zielinska, Janusz Kopczynski, Dariusz Nejc, Radzislaw Kordek, Rafal Sadej, Hanna M. Romanska

**Affiliations:** 1 Department of Pathology, Chair of Oncology, Medical University of Lodz, 92-213 Lodz, Poland; marcin.braun@umed.lodz.pl (M.B.); dominika.piasecka@gumed.edu.pl (D.P.); aleksandra.zielinska3@stud.umed.lodz.pl (A.Z.); radzislaw.kordek@umed.lodz.pl (R.K.); 2 Department of Molecular Enzymology and Oncology, Intercollegiate Faculty of Biotechnology, University of Gdansk and Medical University of Gdansk, 80-211 Gdansk, Poland; kamil.mieczkowski@gumed.edu.pl; 3 Department of Biostatistics and Translational Medicine, Medical University of Lodz, 92-215 Lodz, Poland; bartlomiej.tomasik@umed.lodz.pl (B.T.); konrad.stawiski@umed.lodz.pl (K.S.); 4 Department of Surgical Pathology, Holycross Cancer Centre, 25-734 Kielce, Poland; januszko@onkol.kielce.pl; 5 Department of Surgical Oncology, Medical University of Lodz, 93-513 Lodz, Poland; dariusz.nejc@umed.lodz.pl

**Keywords:** breast cancer, FGFR2, ER, PR, PR transcriptional profile, survival

## Abstract

**Simple Summary:**

FGFR2-ER-PR crosstalk leads to hormone-independent progression of breast cancer. In vitro, FGFR2 stimulates PR transcriptional activity and mediates resistance to anti-ER therapies. The postulated poor prognostic effect of FGFR2 overexpression has not been confirmed at clinical level. Our clinical data show that, counterintuitively, low expression of FGFR is linked to poor prognosis in breast cancer and its prognostic value is dependent on the hormonal receptor status, but not PR transcriptional activity. This shows, that the role of FGFR in breast cancer is more complex, which may explain unsatisfactory results of the clinical trials with FGFR inhibitors.

**Abstract:**

Interaction between fibroblast growth factor receptor 2 (FGFR2) and estrogen/progesterone receptors (ER/PR) affects resistance to anti-ER therapies, however the prognostic value of FGFR2 in breast cancer (BCa) remains largely unexplored. We have recently showed in vitro that FGFR2-mediated signaling alters PR activity and response to anti-ER treatment. Herein, prognostic significance of FGFR2 in BCa was evaluated in relation to both ER/PR protein status and a molecular signature designed to reflect PR transcriptional activity. FGFR2 was examined in 353 BCa cases using immunohistochemistry and Nanostring-based RNA quantification. FGFR2 expression was higher in ER+PR+ and ER+PR- compared to ER−PR− cases (*p* < 0.001). Low FGFR2 was associated with higher grade (*p* < 0.001), higher Ki67 proliferation index (*p* < 0.001), and worse overall and disease-free survival (HR = 2.34 (95% CI: 1.26–4.34), *p* = 0.007 and HR = 2.22 (95% CI: 1.25–3.93), *p* = 0.006, respectively). The poor prognostic value of low FGFR2 was apparent in ER+PR+, but not in ER+PR− patients, and it did not depend on the expression level of PR-dependent genes. Despite the functional link between FGFR2 and ER/PR revealed by preclinical studies, the data showed a link between FGFR2 expression and poor prognosis in BCa patients.

## 1. Introduction

The essential involvement of tumor microenvironment (TME) in breast cancer (BCa) progression and resistance to endocrine therapies has solid mechanistic and clinical foundations [1,2,3]. The key components of TME, i.e., cancer-associated fibroblasts and tumor-infiltrating immune cells, modulate intracellular pathways of BCa through direct or paracrine interactions [1,2,3]. In particular, fibroblast growth factor receptor 2 (FGFR2) has emerged as a principal transducer of signals between TME and ER/PR pathways [4,5].

We and others have shown mechanistically that FGFR2 promotes hormone-independent tumor growth and resistance to endocrine therapies [4,5]. Activation of FGFR2 in BCa cell lines abrogated stimulating effect of estrogen on ER, while decreased FGFR2 expression enhanced cell responsiveness to estrogen [6,7]. In mice, hormone-independent tumors had higher FGFR2 expression and more abundant FGF2-secreting cancer-associated fibroblasts, compared to hormone-dependent tumors [8]. FGFR2 promoted hormone-independent BCa growth also through MAPK or PI3K/AKT-mediated phosphorylation of PR (noncanonical activation of PR), resulting in increased activity of PR, followed by its rapid degradation [8,9,10,11]. Thus, mistakenly deemed as lost, hyperactive PR may drive enhanced BCa cells proliferation and survival via ligand-independent transcriptional activation of PR-targeted genes [12]. In luminal BCa cell lines, FGFR2-mediated deregulation of both ER and/or PR signaling was shown to lead to poor response to treatment with fulvestrant or tamoxifen [13,14,15]. This implies, that FGFR2-mediated noncanonical activation of PR might result in its undetectability on the protein level, leading to false classification of the ‘PR-hyperactive’ tumors as PR-negative [16,17].

In the clinical setting, the postulated poor prognostic effect, likely to result from FGFR2/ER/PR-mediated resistance to endocrine therapies, was analyzed recently in advanced BCa patients, for whom activating genetic alterations in FGFR2 gene (mostly point mutations) were linked to resistance to anti-ER therapies [18,19,20,21,22,23,24,25]. These studies, for the most part inconclusive, involved analyses of genetic alterations of different FGFs or FGFRs genes in metastatic BCa patients subjected to various intensive therapeutic regimens, including those with developed resistance to endocrine therapy. Analyses of a relationship between expression of FGFR2 protein, breast cancer hormone receptor status, and disease outcome also provided inconsistent results [26,27]. Given the documented interactions between FGFR2 and PR, evaluation of the prognostic value of FGFR2 in BCa should account not only for their protein levels, but also for the molecular signature of PR transcriptional activity and rapid turnover [9,17,28,29].

In summary, available data from the clinic do not fully support the anticipated prognostic value of FGFR2 expression in BCa, which partially may be due to the limited insight into FGFR2-mediated noncanonical activation of PR signaling. Herein, we addressed this issue by investigating tumoral tissue from BCa patients for prognostic significance of FGFR2 expression in relation to both hormonal receptor status and a molecular signature designed to reflect PR transcriptional activity in the context of routinely evaluated clinicopathological BCa features.

## 2. Results

### 2.1. Low FGFR2 Expression Is Associated with Unfavorable Clinicopathological Characteristics Including Negative Hormonal Receptor Status

**Figure 1:**
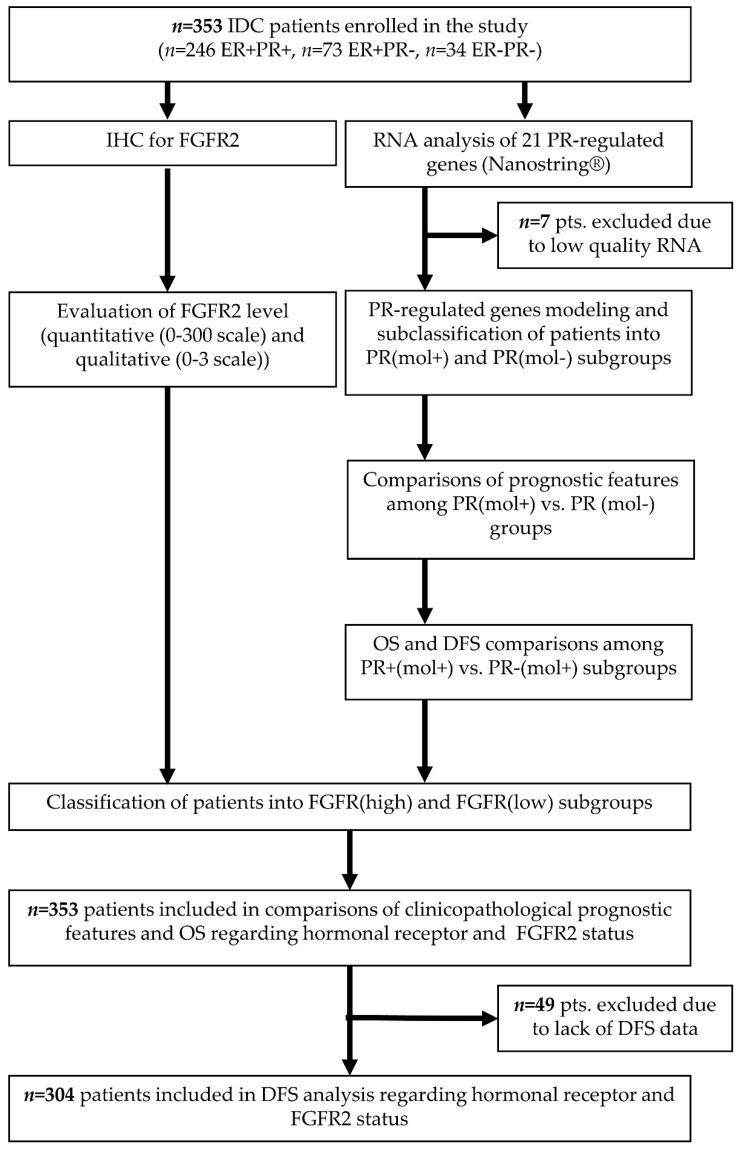
Flowchart of the study design with indication of numbers of patients from the original cohort included in every analysis. IDC—invasive ductal carcinoma, ER—estrogen receptor protein status, PR—progesterone receptor protein status, IHC—immunohistochemistry, FGFR2—fibroblast growth factor receptor 2 protein, PR(mol)—molecular signature progesterone receptor-dependent genes, OS—overall survival, DFS—disease-free survival.

All 353 tumors were qualified for FGFR2 staining, and 346 (98.0%) were of satisfying quality for RNA analyses (Figure 1). Thirty-four (9.6%) patients were ER−PR−, 73 (20.7%) ER+PR-, and 246 (69.7%) ER+PR+, and there were no ER−PR+ cases. ER−PR− and ER+PR− groups displayed poor prognostic features as compared to the ER+PR+ subtype, i.e., higher grade (*p* < 0.001) and Ki67 proliferation index (AKW *p* = 0.011, post-hoc *p* = 0.062 ER+PR+ vs. ER+PR− and *p* = 0.086 ER+PR+ vs. ER−PR−), larger tumor size (AKW *p* < 0.001, post-hoc *p* = 0.297 ER+PR+ vs. ER+PR− and *p* = 0.007 ER+PR+ vs. ER−PR−), and more frequent HER2 amplification (*p* < 0.001) (Table 1).

**Figure 2:**
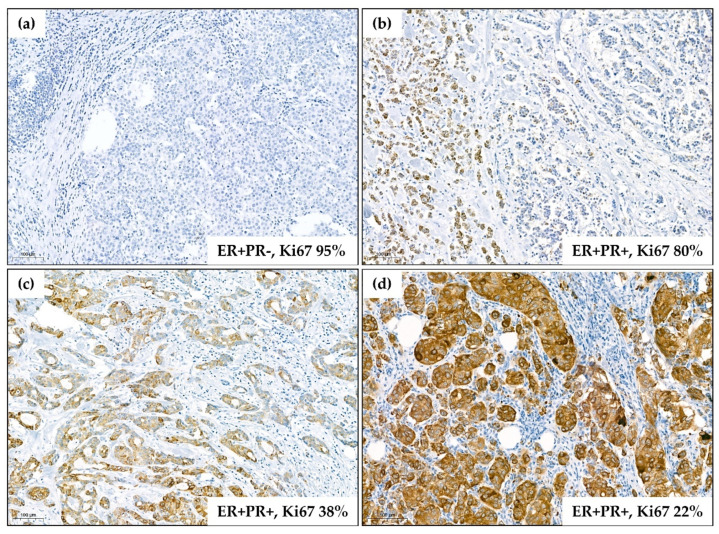
Representative images of immunohistostaining for FGFR2 using H00002263-M01 antibody (Abnova). Corresponding data on hormonal receptor status and Ki67 proliferation index are presented for each tumor. The images are representative for the main observations on the links between FGFR2 levels and clinicopathological features (low levels of FGFR2 associated with ER/PR negativity and high Ki67 index). (**a**) Negativity of cancer cells for FGFR2 (0/3 in semiquantitative scale with H-score of 0/300); (**b**) weak positivity for FGFR2 (1/3 in semiquantitative scale with H-score of 80 (5 × 3 + 30 × 2 + 5 × 1 + 60 × 0)); (**c**) moderate positivity for FGFR2 (2/3 in semiquantitative scale with H-score of 170 (20 × 3 + 35 × 2 + 40 × 1 + 5 × 0)); (**d**) very strong positivity for FGFR2 (3/3 in semiquantitative scale with H-score of 290 (90 × 3 + 10 × 2)). Scale bars indicating 100 µm are applied on each image.

**Figure 3:**
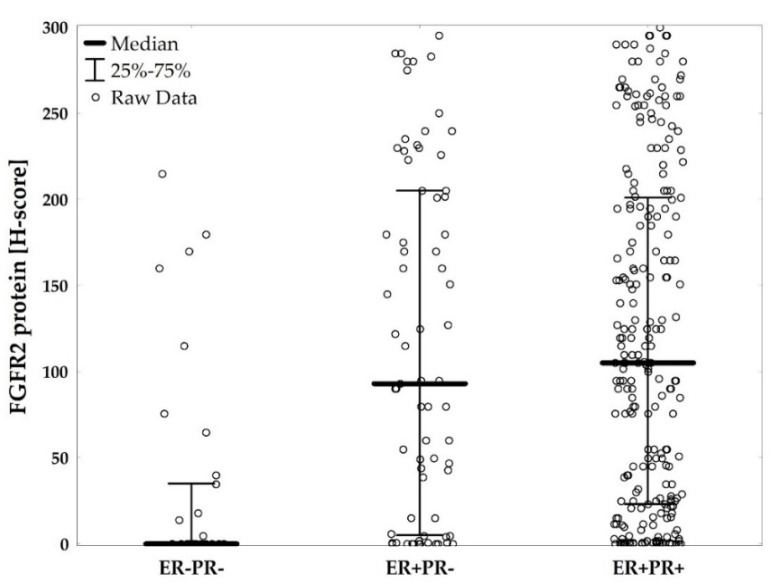
FGFR2 protein levels (H-score) compared between hormonal receptor status subgroups (ER−PR− vs. ER+PR− vs. ER+PR+), *p* < 0.001. *p*-value from Kruskal–Wallis ANOVA test.

FGFR2 protein levels ranged from 0 to 300, and the median level was 95.0 (IQR: 12.0–195.0). There were 152 (43.1%) negative/weakly positive (Figure 2a), 73 (20.7%) moderately positive (Figure 2b), 62 (17.6%) strongly positive (Figure 2c), and 66 (18.7%) very strongly positive cases (Figure 2d). FGFR2 protein levels were significantly lower in ER−PR− versus ER+PR+, and in ER−PR− versus ER+PR− patients (AKW *p* < 0.001 with post-hoc *p* < 0.001 for both comparisons, Figure 3, Table S1). These differences were maintained for FGFR2 gene mRNA (*p* = 0.049 with post-hoc *p* = 0.057 and *p* = 0.432, respectively, Figure S1, Table S1). Expression levels of neither FGFR2 protein nor mRNA showed significant differences between ER+PR− and ER+PR+ tumors (Figure 3 and Figure S1, Table S1).

For clinicopathological and survival analyses patients were dichotomized into FGFR2low and FGFR2high groups by 1st tercile of protein levels (H-score). FGFR2low patients were characterized by a higher both Ki67 proliferation index (*p* = 0.014) and grade (*p* < 0.001), as well as more frequent ER− and PR−negativity (*p* < 0.001), than those with FGFR2high tumors (Table 2).

### 2.2. Low FGFR2 Protein Is Associated with Poor Overall and Disease-Free Survival

**Figure 4:**
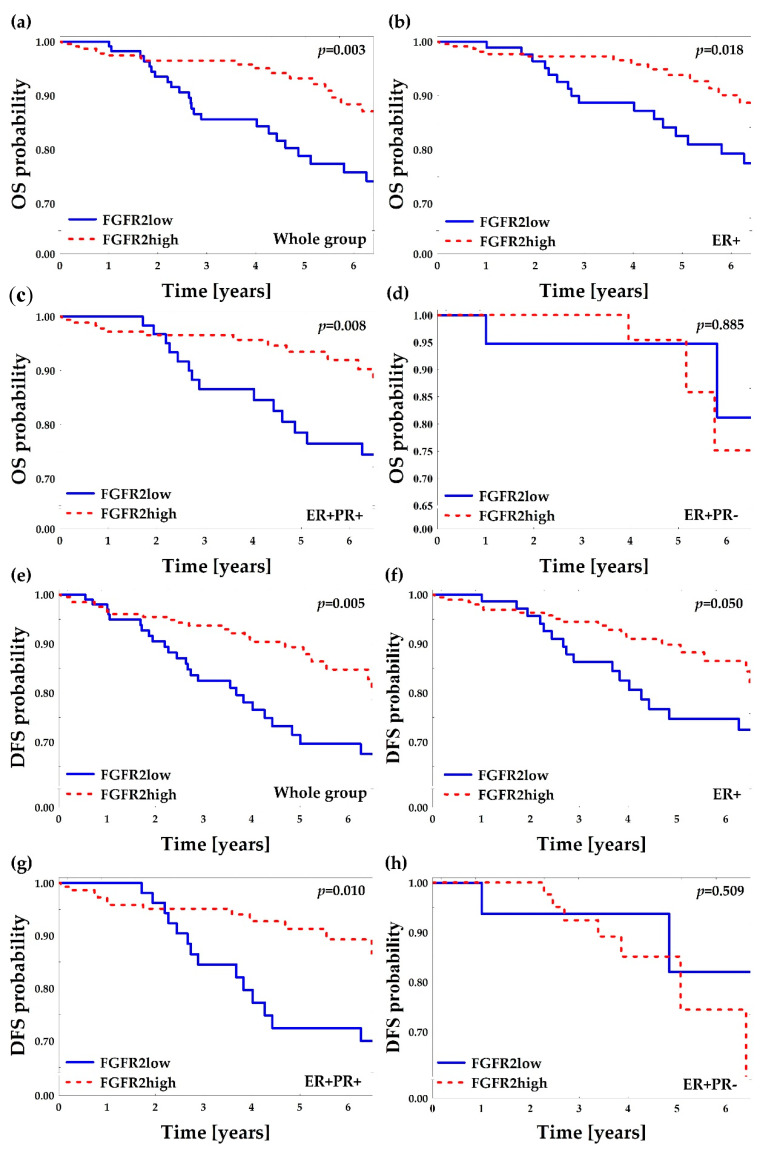
Kaplan–Meier curves for overall survival (OS) and disease-free survival (DFS) probability regarding FGFR2low and FGFR2high patients (divided by 1st tercile of FGFR2 H-score). “ER+” subgroup included all ER+ patients regardless of PR status and it comprised ER+PR+ and ER+PR− subgroups. *p*-values were calculated using log-rank test. (**a**) OS in the whole group; (**b**) OS in the ER+ group; (**c**) OS in ER+PR+ group; (**d**) OS in ER+PR− group; (**e**) DFS in the whole group; (**f**) DFS in the ER+ group; (**g**) DFS in the ER+PR+ group; (**h**) DFS in the ER+PR− group.

Median follow-up of the analyzed group was 4.2 years (IQR: 2.9–6.5) and 41 (11.6%) deaths were recorded. In the univariate analyses of overall survival probability, the following clinicopathological features were associated with poor prognosis: ER−PR−, older age, large size of tumors, presence of lymph node metastases, and advanced disease (Stage IIIB-IV) (Table 1). FGFR2low patients displayed significantly worse overall survival comparing to FGFR2high patients (HR 2.34 (95% CI: 1.26–4.34); log-rank *p* = 0.003, Figure 4a, Table 2 and Table 3). The poor prognostic impact of FGFR2low status was present in ER+ and in ER+PR+, but not in ER+PR− patients (Figure 4b–d). In the multivariate analysis, FGFR2low showed poor prognostic impact on overall survival regardless of the hormonal receptor status (HR = 2.09 (95% CI: 1.08–4.04)), but it was not significant when adjusted to other significant variables from the univariate analyses (only age at diagnosis maintained its significance, Table 3 and Table S2).

Reliable follow-up data for disease-free survival was available for 304 (86.1%) of patients and the median disease-free survival was 3.9 years (IQR: 2.7–6.4) with 47 (15.4%) events in the group. The poor prognostic impact on DFS was displayed for age, high grade, negative hormonal receptor status, larger tumor size, and lymph node metastases (Table S2). FGFR2low patients displayed poorer DFS when compared to FGFR2high patients and this effect was significant for all patients (*p* = 0.005, Figure 4e), ER+ patients (*p* = 0.050, Figure 4f) and ER+PR+ patients (*p* = 0.010, Figure 4g), while nonsignificant for ER+PR− patients (*p* = 0.509, Figure 4h). In the multivariate analysis, FGFR2low showed poor prognostic impact on DFS regardless of the hormonal receptor status (HR = 1.92 (95% CI: 1.03–3.56)) but, as for OS, the effect was not significant when adjusted to other significant DFS variables from the univariate analyses (Table 3 and Table S2).

### 2.3. Subclassification of ER+ Patients by PR(mol) Supports Association of PR-Negativity with Unfavorable Clinicopathological Characteristics

To account for “false” negative PR tumors with undetectable PR at the protein level (due to hormone-independent activation and a rapid turnover of PR), but with effective PR transcriptional activity, a molecular signature reflecting activation of PR-dependent genes (PR(mol)-not PR protein/mRNA status) was developed [12,17,29]. Identification of the ‘signature genes’ was based on the bioinformatic findings of the differences in RNAseq expression levels between ER+PR+, ER+PR−, and ER−PR− BCa cases (ER−PR+ BCa category, as extremely rare and controversial, was not included), which were supported by reported in the literature functional associations between PR and its target genes [12,17,29]. RNA expression of 19 gene-candidates for the PR(mol) was evaluated: *ACOT6*, *BIRC3*, *CEPBD*, *EP400*, *F3*, *FKBP5*, *GAS6*, *HSD11B2*, *KLF4*, *NEDD4*, *NET1*, *RASGRP4*, *RASSF2*, *RGS2*, *S100*, *SIAH2*, *SLC39A14*, *STAT5A*, *UCK2*, the measurement of which was complemented by assessment of *PGR* and *FGFR2* and three housekeeping genes. The list of genes together with the criteria for inclusion are presented in the Table S3.

For four genes’ (*SIAH2*, *PGR*, *BIRC3*, *UCK2*) differences in mRNA level were significantly dependent solely on the PR-status (the essential criterion for the selection), i.e., mRNA of these genes varied significantly between ER+PR+ versus ER+PR−, and between ER+PR+ versus ER−PR− cases, but not between ER+PR− versus ER−PR− patients (used as a reference for lack of PR activity). The tree (joining) hierarchical clustering confirmed strong correlation between *PGR* and *SIAH2* mRNA (Figure S2). Next, k-means clustering was used to design gene signature deemed to reflect activity of PR. Thus, the final PR(mol) involved expression of four genes (*SIAH2*, *PGR*, *BIRC3*, *UCK2*). Reclassification of the whole group by the PR(mol) status showed that no patients with ER−PR− BCa were allocated PR(mol+) (Table 4, Figure 1), implying potential functional significance of PR(mol) only in ER+ patients (Table 4). Accordingly, ER+ BCa (both ER+PR+ and ER+PR−) patients were further subclassified into PR(mol+) and PR(mol−) categories characterized by elevated or decreased expression levels of signature genes, respectively. This resulted in 10 (13.7%) ER+PR− patients described as PR(mol+) and 47 (19.4%) ER+PR+ patients as PR(mol−) (Table 4).

PR(mol+) and PR(mol−) tumors were analyzed in relation to the pathological and clinical features (Table S4). The results showed associations between poor prognostic features and allocation into PR(mol−) subgroup. PR(mol−) tumors were characterized by higher grade (*p* = 0.039) and Ki67 index (*p* = 0.003), more frequent HER2 amplification (*p* = 0.002), and larger size (*p* = 0.007) when compared to PR(mol+) patients. In survival analyses, PR(mol−) patients did not display poorer OS or DFS in comparison to PR(mol+) patients (*p* = 0.551 and *p* = 0.354, respectively, Table S4).

### 2.4. PR(mol) Status Does Not Affect Prognostic Value of FGFR2

**Figure 5:**
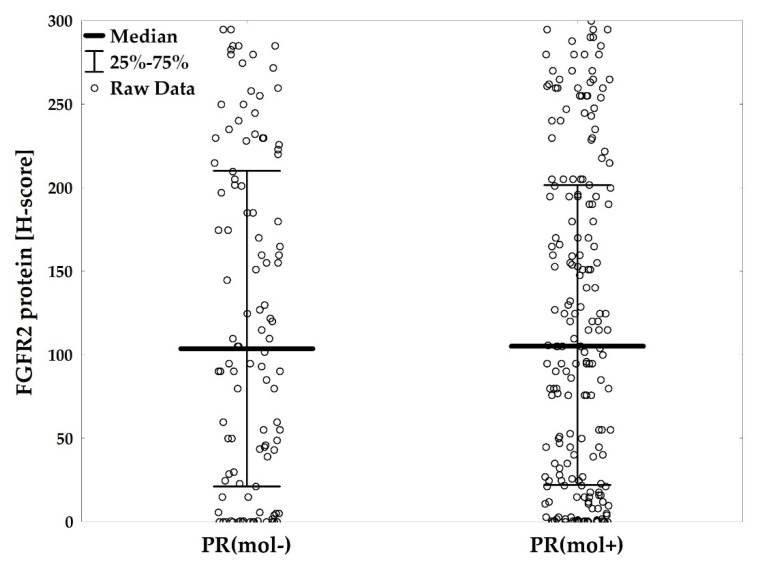
FGFR2 protein levels (H-score) compared regarding progesterone receptor molecular activity status (PR(mol−) vs. PR(mol+)), *p* < 0.001. *p*-values from Kruskal–Wallis ANOVA test.

FGFR2 protein level did not differ significantly between PR(mol+) and PR(mol−) patients (*p* = 0.739), while FGFR2 gene mRNA was significantly higher in PR(mol+) than PR(mol−) cases (*p* = 0.008, Figure 5 and Figure S3, Table S5). Associations of FGFR2low with high grade and high Ki67 proliferation index, described above, were independent of PR(mol) (Table S6). Similarly, poor prognostic impact of FGFR2low on DFS and OS was found not to depend on PR(mol) (Table S6).

### 2.5. Low FGFR2 Gene mRNA Level Is Associated with Poor Overall and Relapse-Free Survival—In Silico Confirmation

**Figure 6:**
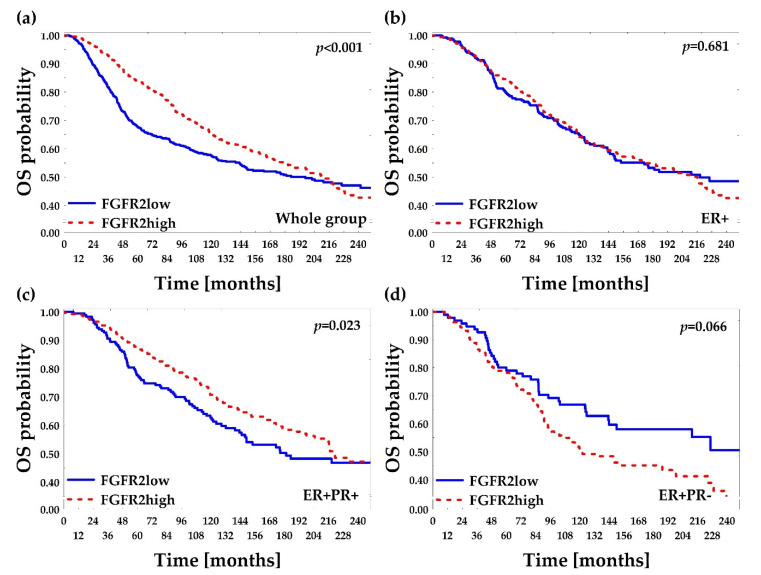
Kaplan–Meier curves for overall survival (OS) probability regarding levels of FGFR2 gene mRNA. METABRIC-TCGA data were accessed from cBioPortal. Invasive ductal carcinoma of no special type samples were divided into FGFR2low and FGFR2high subgroups by 1st tercile of FGFR2 gene mRNA (1st tercile for FGFR2low, 2nd and 3rd terciles for FGFR2high patients). “ER+” subgroup included all ER+ patients regardless of PR status and it comprised ER+PR+ and ER+PR− subgroups. Due to long observation *p*-values were calculated using Gehan’s Wilcoxon test. (**a**) OS in the whole group (*n* = 1242); (**b**) OS in the ER+ group (*n* = 1070); (**c**) OS in the ER+PR+ group (*n* = 720); (**d**) OS in the ER+PR− group (*n* = 350).

Prognostic significance of FGFR2 was verified in silico. Out of 2509 BCa patients from the METABRIC-TCGA database [30,31], 1242 fulfilled the requirements of the study, i.e., invasive ductal carcinoma of no special type (IDC, NST), available data on follow-up, FGFR2 gene mRNA, and hormonal receptor status, and were selected for the analysis (Figure S4). Overall survival was compared between FGFR2low versus FGFR2high patients (divided by 1st tercile of FGFR2 gene mRNA, Figure 6a–d). The poorer OS of FGFR2low than FGFR2high cases was apparent in all patients and in the ER+PR+ subgroup (Gehan–Wilcoxon *p* < 0.001, Figure 6a and *p* = 0.023 Figure 6c, respectively), supporting our data presented in Figure 4. However, in the ER+PR− group, FGFR2high patients tended to display poorer OS than FGFR2low cases (Gehan–Wilcoxon *p* = 0.066, Figure 6d). A possible overwriting effect of FGFR2 gene alterations (especially activating mutations/amplification) on poor survival was considered by accession for data on genetic alterations. However, FGFR2 gene amplification or point mutation were found in only 24 out of 1636 (1.47%) invasive ductal carcinoma of no special type (IDC, NST) patients and had no effect on overall survival (log-rank *p* = 0.170). No DFS data were accessible through the TCGA database.

In a complementary approach, an impact of FGFR2 gene mRNA levels (microarray data) on OS of 1402 BCa patients (other than considered in the TCGA database, Figure S4) meeting inclusion criteria of this study was estimated by the Kaplan–Meier plotter [32]. The FGFR2low patients (<1st tercile of mRNA level) showed worse overall survival compared to FGFR2high patients (HR 1.82 (95% CI: 1.40–2.40), *p* < 0.001; Figure S5a). This poor prognostic effect was mostly apparent for ER+ patients (HR 1.96 (95% CI: 1.30–3.00), *p* < 0.001; Figure S5b), while PR status did not affect OS in the group (likely due to low number of cases with reported PR status, Figure S5c,d). DFS data along with FGFR2 gene mRNA from the same database was accessible for 3951 BCa cases. Similarly to OS, FGFR2low patients displayed poor prognostic effect when compared to FGFR2high patients (HR 1.54 (95% CI: 1.30–1.80), *p* < 0.001, Figure S5e). This effect was also apparent in both ER+ and ER+PR+ subgroups (*p* < 0.001 and *p* = 0.002, respectively, Figure S5f–g), but not in ER+PR− patients (*p* = 0.363, Figure S5h).

## 3. Discussion

In contrast to the reported functional association between FGFR2 and resistance to anti-ER treatment in BCa, our results suggested that lack or low expression of FGFR2 is characteristic of hormone receptor-negative and poorly differentiated tumors, and is prognostic for poor survival, regardless of the transcriptional activity of PR. This finding was confirmed across two external open access databases encompassing almost 5000 BCa patients, as well as in the multivariate analyses.

The deleterious crosstalk between FGFR2 and ER/PR signaling in BCa has been robustly documented in mechanistic studies, but supporting observations from the clinic are still lacking [4,5]. FGFR2 acts as an essential regulator of steroid hormone receptors activity by several, likely independent mechanisms, i.e., interaction with ER (shifting ER binding to DNA) and/or hormone-independent activation and rapid degradation of the receptors [5,9,15]. Noncanonical hyperactivation of PR and alteration of its molecular communication with ER (reviewed in [5]) have been shown to strongly promote hormone independence and resistance to anti-ER therapies. These effects are associated with a rapid turnover/degradation of the PR protein, which is undetectable by immunohistochemistry, routinely used to assess ER/PR protein levels (“concealed positivity”) [13,14,15]. These were the premises for the presented analyses of the prognostic value of FGFR2 expression in the context of not only ER and PR protein status, but also PR transcriptional activity.

In contrast to the postulated relationship between high FGFR2 and poor prognosis, we found that worse overall and disease-free survival of BCa patients was associated with low FGFR2 expression on both protein and mRNA levels. Moreover, low FGFR2 was also associated with prognostically unfavorable tumor characteristics, i.e., high proliferation index and poor differentiation, which may suggest that in certain biological settings, tumor aggressiveness might be featured or enforced by low FGFR2. Although there has been no clear mechanistic explanation for this adverse effect of FGFR2 loss, available data support a differential, context-dependent prognostic value of FGFR2. For example, high FGFR2 expression has been recently shown to correlate with increased BCa sensitivity to endocrine therapy combined with inhibitors of CDK4/6 [33]. Loss of function mutations in FGFR2 gene contributed to melanoma progression, whilst gain of function alterations were reported to promote growth of endometrial carcinoma [34,35,36]. Conditional FGFR2 gene knockout and low or reduced FGFR2 expression were linked with increased sensitivity to chemically induced squamous cell carcinoma of the skin [37], more aggressive growth of hepatocellular carcinoma [38], and increased epithelial-to-mesenchymal transition [39].

Our results confirmed the tight link between FGFR2 expression and the ER/PR status, showing that low FGFR2 is characteristic of ER−PR− tumors. Furthermore, the poor prognostic effect of low FGFR2 was found to be lost in ER+PR− patients. Although the assumed ‘concealed positivity’ of PR induced by FGFR2 has a solid biological background [18,19,20], our data fail to support it. The designed signature deemed to reflect transcriptional activity of PR and, when used for subclassification of patients with ER+ tumors, maintained poor prognostic effect of PR−negativity. However, the impact of FGFR2 on patients’ survival was found to be independent of the status determined by the PR signature. This may suggest that a functional relationship between FGFR2 and PR is likely to be influenced by additional factors, including interaction with ER, identification of which in vivo remains notoriously difficult and requires much more comprehensive molecular evaluation.

The major limitation of our clinical analyses concerns incomplete data on relapse and progression in 15% of patients of the study group. Even though the guidelines for BCa treatment care are consistent throughout the EU, many patients undergo specific procedures at different locations, which significantly hinders identification of progression and relapses. Of note, distribution of hormonal receptor subgroups in our cohort was similar to the worldwide reports [40]. The external validation of our findings in the TCGA and KM-plotter databases, which accounted for FGFR2 gene mRNA and importantly for genetic alterations in FGFR2 gene, diminished the possible effect of this limitation.

## 4. Materials and Methods

### 4.1. Patient Selection and Collection of Histopathological and Clinical Data

Formalin-fixed, paraffin-embedded (FFPE) tumoral tissue of invasive breast carcinoma, of no special type (IDC, NST) was collected from 353 patients treated at the Regional Oncologic Centre of Copernicus Memorial Hospital, Lodz, Poland and at the Holycross Cancer Centre, Kielce, Poland, between 2004 and 2018. Initial diagnoses were confirmed on hematoxylin/eosin (H&E) stained sections. Patient characteristics (in accordance with the WHO 2012 and 2019 classification of BCa [41]) are presented in Table 1. Statuses of ER and PR were determined according to the Allred scoring system [42]. “ER+” subgroup included all ER+ patients regardless of PR status and it comprised ER+PR+ and ER+PR− subgroups. HER2 status was defined using Herceptest™ (Agilent, Santa Clara, CA, USA) and FISH, whereas Ki67 index was assessed according to the guidelines applicable at the time of diagnosis [43,44]. The study was approved by the Local Research Ethics Committee (No. RNN/34/16/KE).

### 4.2. Immunohistochemistry for FGFR2

Immunohistochemical staining (IHC) for FGFR2 in all tumors was conducted using a mouse monoclonal anti-FGFR2 antibody (H00002263-M01; clone 1G3, Abnova, Heidelberg, Germany) (Figure 2). To confirm specificity of the staining, additional IHC with a mouse anti-FGFR2 antibody (Sc-6930, Santa Cruz, CA, USA) was performed in randomly selected samples. Following manufacturer’s recommendations, tissue samples of gastric adenocarcinoma and lymph node were used as positive and negative controls for IHC, respectively. Immunohistochemical procedures were carried out on 5-µm paraffin sections, as reported previously [9,15]. All slides were digitalized using Pannoramic 1000 Scanner (3DHistech, Sysmex, Kobe, Japan). FGFR2 levels were quantified according to the semiquantitative H-score approach by two independent pathologists (MB, HR). The data were presented in 0–300 scale resulting from multiplication of percentage of positive cells by intensity of staining: 0—no staining, 1–3—increased intensity of both cytoplasmic and membrane staining (subgroups by H-score: 0–75 for negative/weak; 76–150 for moderate; 151–225 for strong; 226–300 for very strong expression) (Figure 2). Cases from 1st tercile of H-score were regarded as FGFR2low and cases from 2nd and 3rd terciles were classified as FGFR2high.

### 4.3. RNA Quantification

For RNA quantification, representative tumoral FFPE samples from areas with no necrosis, fibrosis, or calcification were selected and dissected. RNA was isolated using RNeasy FFPE Kit (Qiagen, Hilden, Germany) followed by quality control on Tapestation 2200 (Agilent, Santa Clara, CA, USA). Quantification of RNA was done by Nanostring^®^ company using nCounter PlexSet Expression analysis (Seattle, WA, USA) [45,46]. RNA counts were normalized using nSolver^®^ Analysis package (Nanostring, Seattle, WA, USA). Four negative controls (normal breast gland) and 16 internal controls (two samples of the same tumor; *n* = 5 and RNA measurement in duplicates, *n* = 3) were applied.

### 4.4. In Silico Analysis of TCGA Data

The Cancer Genome Atlas (TCGA) Research Network (https://www.cancer.gov/tcga) was accessed for RNAseq data of BCa samples with both ER and PR statuses reported. The counts were normalized for GC-content effect and gene length using less robust local regression, global-scaling, and full-quantile normalization. The first quantile (0.25) mean across all samples was used as the threshold in filtering transcripts. Differential expression analysis between ER+PR+ and ER−PR− patients was performed by fitting the linear model and computation moderated t-statistics, moderated F-statistic, and log-odds of differential expression by empirical Bayes moderation of the standard errors towards a common value. Based on *p*-values, genes/candidates with the most significant differences in the level of expression were identified and, considered to reflect transcriptional activity of PR, were collectively named ‘the PR−signature’ (called PR(mol)). R software was utilized in the analysis (packages: TCGAbiolinks, EDASeq, limma). Relevant clinical and pathological data were matched to allow comparative analysis of the findings from our study group with other representative datasets.

### 4.5. Statistical Analysis

Continuous data was presented as medians with interquartile ranges (IQR), whereas nominal data as numbers followed by percentages in brackets. After evaluation of distribution’s normality using the Shapiro–Wilk test, continuous variables were compared by the Mann–Whitney *U*-test for two groups or the Kruskal–Wallis test (AKW; with Conover–Inman post-hoc test) for multiple groups, in case of non-normal distribution. In case of normal distribution, Student’s *t*-test or one-/two-way block ANOVA (with Tukey’s post-hoc test) were used. Differences between categorical variables were evaluated using the Pearson’s chi-squared test. The Spearman’s rank correlation coefficients were calculated for correlations. Benjamini–Hochberg (BH) correction in case of multiple comparisons was applied. For development of PR−signature, k-means clustering with Euclidean distances between clusters, and hierarchical clustering with 1-R for linkage distance reporting were applied. Disease-free survival (DFS, the time from surgery to relapse, progression or death with censoring of living patients) and overall survival (OS, the time from diagnosis to death with censoring of living patients) were presented using Kaplan–Meier curves, and compared using the log-rank test unless noted otherwise [47]. A multivariate analysis of OS and DFS was performed using Cox’s proportional hazard regression models. The Statistica 13.1 package (Dell Inc., Round Rock, TX, USA) was used. *p*-values < 0.05 were considered as statistically significant.

## 5. Conclusions

In summary, our data show that, in contrast to the previously reported link between increased activity of FGFR2 and resistance to anti-ER therapies, low expression of FGFR2 is associated with poor prognosis in BCa. These findings indicate a multifactorial regulation of the FGFR2-ER/PR crosstalk and may provide, at least partially, an explanation for unsatisfactory results of clinical trials of FGFR inhibitors in BCa as well as open new avenues for the study of complexity of FGFR2 role in BCa pathogenesis.

## Figures and Tables

**Table 1:** Pathological and clinical characteristics of the study group regarding immunohistochemistry-based hormonal receptor status. Nominal variables are presented as raw values followed by percentages of the eligible groups, and continuous variables are presented as medians and interquartile ranges in brackets.

Variable	Whole Group *n* = 353 (100)	ER−PR− *n* = 34 (9.6)	ER+PR− *n* = 73 (20.7)	ER+PR+ *n* = 246 (69.7)	*p*-Value
Age (years) ^1^	63.8	63.6	64.7	63.6	0.132
	(55.0–71.3)	(55.7–73.1)	(60.0–70.6)	(52.7–70.8)	
Menopausal status ^2^					0.063
Pre	37 (11.4)	2 (6.1)	3 (4.6)	32 (14.1)	
Post	288 (88.6)	31 (93.9)	62 (95.4)	195 (85.9)	
Grade ^2^					<0.001 *
1	42 (11.9)	2 (5.9)	9 (12.3)	31 (12.6)	
2	227 (64.3)	12 (35.3)	43 (58.9)	168 (69.9)	
3	84 (23.8)	20 (58.8)	21 (28.8)	42 (17.5)	
Ki67 (%) ^1^	18.0	32.0	20.0	12.0	0.011 *
	(5.0–30.0)	(30.5–35.5)	(10.0–40.0)	(5.0–25.0)	
HER2 amplification positivity ^2^	45 (12.7)	12 (35.3)	13 (17.8)	20.0 (8.1)	<0.001 *
Tumor size (mm) ^1^	20.0	25.0	25.0	20.0	0.004 *
	(15.0–27.0)	(15.0–35.0)	(19.0–30.0)	(15.0–25.0)	
T feature ^2^					0.064
pT1	143 (53.0)	13 (40.6)	15 (38.5)	115 (57.8)	
pT2	116 (43.0)	16 (50.0)	22 (56.4)	78 (39.2)	
pT3-4	11 (4.0)	3 (9.4)	2 (5.1)	6 (3.0)	
Metastases present ^2^	116 (33.5)	13 (42.4)	25 (34.7)	77 (32.0)	0.475
N feature ^2^					0.733
pN0	230 (66.5)	17 (57.6)	47 (65.3)	164 (68.0)	
pN1	81 (23.4)	10 (30.3)	16 (22.2)	55 (22.8)	
pN2-3	34 (10.1)	4 (12.1)	9 (12.5)	22 (9.1)	
Staging ^2^					0.289
Very early (IA)	135 (39.1)	11 (34.4)	23 (31.9)	101 (41.9)	
Early (IB-IIIA)	189 (54.8)	17 (53.1)	45 (62.5)	127 (52.7)	
Advanced (IIIB-IV)	21 (6.1)	4 (12.5)	4 (5.6)	13 (5.4)	
Multifocality ^2^	39 (14.3)	3 (8.8)	6 (15.4)	30 (15.0)	0.622
DCIS present ^2^	103 (29.1)	10 (29.4)	23 (31.5)	70 (28.3)	0.871
Hormonotherapy ^2^	220 (81.2)	0 (0.0)	46 (82.1)	174 (86.6)	<0.001 *
Neoadjuvant therapy ^2^	231 (75.5)	26 (89.7)	30 (48.4)	175 (81.4)	<0.001 *
Adjuvant chemotherapy ^2^	150 (62.2)	25 (80.7)	38 (66.7)	87 (56.9)	0.032 *
Adjuvant radiotherapy ^2^	151 (64.8)	11 (50.0)	30 (61.2)	110 (67.9)	0.111
Progression/relapse ^3^	30 (9.8)	10 (31.3)	8 (11.9)	12 (5.8)	<0.001 *
Disease-free survival (years)	3.9 (2.7–6.4)	3.6 (2.2–4.5)	3.8 (2.5–4.9)	4.2 (2.9–6.6)	
Deaths ^3^	41 (11.6)	10 (29.4)	6 (8.2)	25 (10.1)	<0.001 *
Overall survival (years)	4.2 (2.9–6.5)	3.6 (2.6–4.5)	4.0 (2.7–5.5)	4.7 (3.0–6.7)	

^1^ Kruskal–Wallis ANOVA test, ^2^ Pearson’s chi-squared test, ^3^ log-rank test, * significant differences.

**Table 2:** Clinicopathological features in fibroblast growth factor receptor 2 (FGFR2)low versus FGFR2high patients (divided by 1st tercile of H-score protein level). Nominal variables are presented as raw values followed by percentages of the respective groups, continuous variables are presented as medians and interquartile ranges in brackets.

Variable	FGFR2low *n* = 117 (33.1)	FGFR2high *n* = 236 (66.9)	*p*-Value
Age (years) ^1^	64.6 (56.5–75.2)	63.4 (54.2–69.9)	0.078
Menopausal status ^2^			0.078
Pre	8 (7.1)	29 (13.7)	
Post	104 (92.9)	183 (86.3)	
Grade ^2^			<0.001 *
1	7 (5.9)	35 (14.8)	
2	65 (55.6)	162 (68.6)	
3	45 (38.5)	39 (16.5)	
Ki67 (%) ^1^	23.5 (12.0–40.0)	15.0 (5.0–28.0)	0.014 *
HER2 amplification positivity ^2^	11 (9.4)	33 (14.0)	0.220
Hormonal status ^2^			<0.001 *
ER−PR−	25 (21.4)	9 (3.8)	
ER+PR−	22 (18.8)	51 (21.6)	
ER+PR+	70 (59.8)	176 (74.6)	
PR molecular status ^2^			0.757
PR(mol+)	59 (66.3)	145 (64.4)	
PR(mol-)	30 (33.7)	80 (35.6)	
Tumor size (mm) ^1^	20.0 (15.0–30.0)	20.0 (15.0–25.0)	0.898
T feature ^2^			0.731
pT1	55 (51.4)	88 (54.0)	
pT2	46 (43.0	69 (42.3)	
pT3-4	6 (5.6)	6 (3.7)	
Metastases present ^2^	44 (38.6)	76 (32.9)	0.296
N feature ^2^			0.682
pN0	73 (64.0)	156 (67.5)	
pN1	30 (26.3)	51 (22.1)	
pN2-3	11 (9.6)	24 (10.4)	
Staging ^2^			0.234
Very early (IA)	40 (35.1)	95 (41.1)	
Early (IB-IIIA)	69 (60.5)	119 (51.5)	
Advanced (IIIB-IV)	5 (4.4)	17 (7.4)	
DCIS present ^2^	30 (25.6)	72 (30.5)	0.342
DFS events ^3^	25 (24.8)	22 (10.8)	0.005 *
Disease-free survival (years)	3.9 (2.6–6.6)	3.9 (2.7–5.8)	
Deaths ^3^	23 (19.7)	18 (7.6)	0.003 *
Overall survival (years)	4.6 (2.7–6.7)	4.1 (2.9–6.4)	

^1^ Mann–Whitney U-test, ^2^ Pearson’s chi-squared test, ^3^ log-rank test, * significant differences.

**Table 3:** Results from Cox multivariate analysis for overall survival (OS) and disease-free survival (DFS) regarding FGFR2 status adjusted for variables significant in the univariate survival analyses.

Outcome	HR (95% CI) Raw	HR (95% CI) Adjusted for Hormonal Status	HR (95% CI) Adjusted for Hormonal Status, Age and Tumor Size
OS (FGFR2high as reference)	2.34 (1.26–4.34), *p* = 0.007	2.09 (1.08–4.04), *p* = 0.028	1.45 (0.73–2.90), *p* = 0.283
DFS (FGFR2high as reference)	2.22 (1.25–3.93), *p* = 0.006	1.92 (1.03–3.56), *p* = 0.038	1.25 (0.66–2.37), *p* = 0.496

**Table 4:** Reclassification of cases using the PR-molecular signature (PR(mol)). Data presented as numbers and percentages in brackets. ER—estrogen receptor status, PR—progesterone receptor; *p* < 0.001, Pearson’s chi-squared test.

Hormonal Status	PR(mol+)	PR(mol−)
ER−PR−	0 (0.0%)	32 (100.0%)
ER+PR−	10 (13.7%)	63 (86.3%)
ER+PR+	194 (80.6%)	47 (19.4%)
All	204 (59.1%)	142 (40.9%)

## Supplementary Materials

The following are available online at https://www.mdpi.com/2072-6694/12/9/2713/s1. Figure S1: FGFR2 mRNA levels compared between hormonal receptor status subgroups (ER−PR− vs. ER+PR− vs. ER+PR+), *p* = 0.049 from ANOVA test, Figure S2: Hierarchical tree-clusterization of genes included in PR−dependent molecular signature. All patients with good quality RNA were included in this analysis. Linkage distance is showed as 1-R (Spearman), Figure S3: FGFR2 mRNA levels compared between estrogen receptor status and progesterone receptor molecular activity status (ER−PR (mol−) vs. ER+PR (mol−) vs. ER+PR (mol+)), *p* = 0.002. *p*-value from ANOVA test, Figure S4: Flowchart of the in silico verification with indication of numbers of patients from the external databases included in every analysis. “ER+” subgroup included all ER+ patients regardless of PR status and it comprised ER+PR+ and ER+PR− subgroups. IDC, NST—invasive ductal carcinoma of no special type, ER—estrogen receptor protein status, PR—progesterone receptor protein status, FGFR2—fibroblast growth factor receptor 2 protein, Figure S5: Kaplan–Meier curves for OS (a–d) and PFS (e–h) regarding FGFR2 microarray mRNA levels. “ER+” subgroup included all ER+ patients regardless of PR status and it comprised ER+PR+ and ER+PR− subgroups. FGFR2low stands for 1st tercile and FGFR2high for 2nd and 3rd terciles. Plots were generated using online open access tool KM plotter (encompassing patients different than those included in TCGA database). (a) OS probability in all 1402 breast cancer patients, (b) OS probability in all 548 ER+ breast cancer patients, (c) OS probability in all 76 ER+PR+ patients, (d) OS probability in all 25 ER+PR− patients, (e) PFS probability in all 3951 breast cancer patients, (f) PFS probability in all 2061 ER+ breast cancer patients, (g) PFS probability in all 559 ER+PR+ patients, (h) PFS probability in all 154 ER+PR− patients, Table S1: Expression of FGFR2 protein and mRNA in relation to ER/PR status. Nominal variables are presented as raw values followed by percentages of the respective groups, continuous variables are presented as medians and interquartile ranges in brackets, Table S2: Cox univariate and multivariate overall and disease-free survival analyses according to prognostic clinicopathological features, including FGFR2 status (low vs. high divided by 1st tercile of protein H-score). Hazard ratios are present for nominal variables, while β-parameters for continuous variables. Variables significant only in univariate analyses were incorporated in multivariate analyses. CI—confidence interval, NA—not applicable, Table S3: List of PR−dependent genes (PR (mol)—“molecular signature”) signifying receptor activation and rapid degradation with respective reasons for inclusion, Table S4: Clinical and pathological characteristics of PR (mol−) versus PR (mol+) subgroups within ER+ patients. Nominal variables are presented as raw values followed by percentages of the respective groups, continuous variables are presented as medians and interquartile ranges in brackets, Table S5: Expression of FGFR2 protein and mRNA in PR (mol−) versus PR (mol+) patients within ER+ subgroup. Nominal variables are presented as raw values followed by percentages of the respective groups, continuous variables are presented as medians and interquartile ranges in brackets, Table S6: Multivariate analyses of the combined effect of FGFR2 (protein) status and PR(mol) status on the poor prognostic associations characterized for FGFR2(low). The analysis involves only ER+ patients. Hazard ratios with confidence intervals are present for overall and disease-free survival, β-parameters, and standard deviation for Ki67 proliferation index and odds ratios (OR) with confidence intervals for tumor grade.
