# Supplementary material for: Hormonal Receptor Status Determines Prognostic Significance of FGFR2 in Invasive Breast Carcinoma
Source: Cancers (Basel). 2020 Sep 22;12(9):2713. doi: 10.3390/cancers12092713 (PMC7564845; doi:10.3390/cancers12092713)
Supplement: Supplementary file 1 [file cancers-12-02713-s001.pdf]

cancers

MDPI

Article

Hormonal Receptor Status Determines Prognostic Significance of FGFR2 in Invasive Breast Carcinoma

Marcin Braun, Dominika Piasecka, Bartlomiej Tomasik, Kamil Mieczkowski, Konrad Stawiski, Aleksandra Zielinska, Janusz Kopczynski, Dariusz Nejc, Radzislaw Kordek, Rafal Sadej and Hanna M. Romanska

Table S1. Expression of FGFR2 protein and mRNA in relation to ER/PR status. Nominal variables are presented as raw values followed by percentages of the respective groups, continuous variables are presented as medians and interquartile ranges in brackets;.

Variable	ER-PR-	ER+PR-	ER+PR+	*p*-value
FGFR2 protein [H-score] [1]	0.0 (0.0–26.5)	93.0 (5.0–205.0)	105 (25.0–202.0)	<0.001 *
FGFR2 by H-score quartiles [2]				
0–75	27 (84.4)	31 (42.5)	90 (25.9)	<0.001 *
76–150	2 (6.3)	13 (17.8)	57 (23.5)	
151–225	3 (9.4)	13 (17.8)	45 (18.6)	
226–300	0 (0.0)	16 (21.9)	50 (20.7)	
FGFR2 mRNA [log2] [1]	7.8 (6.6–9.2)	8.5 (7.2–9.4)	8.6 (8.0–9.3)	0.049 *

[1] ANOVA Kruskal-Wallis or ANOVA test, [2] Pearson's chi-squared test, * significant differences.

Table S2. Cox univariate and multivariate overall and disease-free survival analyses according to prognostic clinicopathological features, including FGFR2 status (low vs. high divided by 1st tercile of protein H-score). Hazard ratios are present for nominal variables, while β-parameters for continuous variables. Variables significant only in univariate analyses were incorporated in multivariate analyses. CI—confidence interval, NA—not applicable.

Variable	Hazard ratio (95% CI)/ β-parameter (SE) univariate analysis	*p*-value	Hazard ratio/ β-parameter multivariate analysis	*p*-value
**Overall survival**				
Age [years]	0.08 (0.01)	<0.001 *	0.05 (0.02)	0.002 *
Grade [ref. G1]	NA	0.987	NA	NA
Ki67 [%]	0.01 (0.02)	0.583	NA	NA
HER2 [ref. no amplification]	1.29 (0.54–3.07)	0.566	NA	NA
Hormonal receptor status [ref. ER+PR+]	4.18 (1.98–8.84)	<0.001 *	2.17 (0.89–5.29)	0.230
FGFR2 status [ref. high]	2.34 (1.26–4.34)	0.007 *	1.26 (0.63–2.52)	0.518
Tumor size [mm]	0.04 (0.01)	<0.001 *	0.02 (0.02)	0.120
Lymph node metastases [ref. absent]	1.90 (0.99–3.65)	0.052	NA	NA
Stage [ref. very early]	30.80 (2.06–460.49)	0.017 *	1.87 (0.05–67.51)	0.937
**Disease-free survival**				
Age [years]	0.05 (0.01)	<0.001 *	0.03 (0.02)	0.007 *
Grade [ref. G1]	4.31 (1.00–18.52)	0.012 *	3.55 (0.45–27.77)	0.188

Ki67 [%]	0.01 (0.01)	0.949	NA	NA
HER2 [ref. no amplification]	0.92 (0.41–2.05)	0.834	NA	NA
Hormonal receptor status [ref. ER+PR+]	3.88 (1.94–7.73)	<0.001 *	1.63 (0.68–3.90)	0.432
FGFR2 status [ref. high]	2.22 (1.25–3.93)	0.007 *	1.31 (0.65–2.62)	0.448
Tumor size [mm]	0.04 (0.01)	<0.001 *	0.01 (0.01)	0.293
Lymph node metastases [ref. absent]	1.90 (1.04–3.47)	0.036 *	1.49 (0.76–2.93)	0.243
Stage [ref. very early]	2.73 (0.88–8.43)	0.205	NA	NA

*significant differences.

Table S3. List of PR-dependent genes (PR(mol) – "molecular signature") signifying receptor activation and rapid degradation with respective reasons for inclusion.

Target Identifier	Gene name	Reason for inclusion to the signature
NM_000926	*PGR* (progesterone receptor)	Progesterone receptor gene transcript - levels of *PGR* mRNA are strongly correlated with PR protein and its downstream pathway activity [1–4]
NM_022970	*FGFR2* (fibroblast growth factor receptor 2)	Main gene of interest in this study – good marker of PR expression in the TCGA analysis - significantly different levels of FGFR2 in ER+PR+ and ER+PR-
NM_005067	*SIAH2* (Seven in Absentia Homolog 2)	PR degradation marker - an ubiquitin E3 ligase involved in degradation of PR, especially in tumours with high activity of PR pathway [5]
NM_198400	*NEDD4* (Neural Precursor Cell Expressed, Developmentally Down-Regulated 4, E3 Ubiquitin Protein Ligase)	PR degradation marker - a ubiquitin E3 ligase reported to be involved in degradation of PR downstream mediators [6]
NM_001993	F3 (Coagulation Factor III, Tissue Factor)	Marker of PR activity – secreted molecule highly up-regulated (18-fold change) by PR [7–9]; displayed significantly different levels between ER+PR+ and ER+PR- in the TCGA analysis
NM_005195	CEBPD (CCAAT/Enhancer Binding Protein Delta)	PR downstream activation marker – transcription factor up-regulated (6-fold change) by PR [7–9]; displayed significantly different levels between ER+PR+ and ER+PR- in the TCGA analysis
NM_000820	GAS6 (Growth Arrest Specific 6)	PR downstream activation marker – secreted molecule highly up-regulated (23-fold change) by PR [7–9]
NM_005980	S100p (S100 Calcium Binding Protein P)	PR downstream activation marker – calcium-binding protein, up-regulated (2–4 fold change) by PR [7–9]
NM_009061	RGS2 (Regulator of G-protein signalling 2)	PR downstream activation marker – GTPase activating protein, up-regulated in the report by Knutson et al, verified *in vitro* [8], significantly different levels between ER+PR+ and ER+PR- in the TCGA analysis
NM_001037162	ACOT6 (Acyl-CoA Thioesterase 6)	PR downstream activation marker – involved in metabolism, up-regulated by PR [8]
NM_004235	KLF4 (Kruppel Like Factor, Epithelial Zinc Finger Protein EZF)	PR downstream activation marker – transcription factor up-regulated (6–8 fold change) by PR [7–9]

NM_000196	HSD11B2 (Hydroxysteroid 11-Beta Dehydrogenase 2)	PR downstream activation marker – involved in cholesterol or steroid metabolism and trafficking, highly up-regulated (23-8 fold change) by PR [7–9]
NM_003152	STAT5A (Signal Transducer And Activator Of Transcription 5A)	PR downstream activation marker – transcription factor up-regulated (6 fold change) by PR [7–9]
NM_014737	RASSF2 (Ras Association Domain Family Member 2)	PR downstream activation marker – involved in signal transduction from membrane, up-regulated (10 fold change) by PR, verified *in vitro* [7–9]
NM_001165	BIRC3 (Baculoviral IAP Repeat Containing 3)	PR downstream activation marker – involved in cell cycle and apoptosis, up-regulated (7 fold change) by PR [7–9]
NM_170604	RASGRP4 (RAS Guanyl Releasing Protein 4)	PR downstream activation marker – selected from the TCGA analysis as one of the most significantly different between PR+ and PR- patients, member of RAS guanyl nucleotide-releasing, up-regulated by PR [8]
NM_015409	EP400 (E1A Binding Protein P400)	PR downstream activation marker – the upregulation effect [7–9]; significantly different levels between ER+PR+ and ER+PR- in the TCGA analysis
NM_001145777	FKBP5 (FK506 Binding Protein 5)	PR downstream activation marker – involved in chaperones/protein folding up-regulated (3–9 fold change) by PR [7–9]
NM_001047160	NET1 (Neuroepithelial Cell Transforming 1, ARHGEF8)	PR downstream activation marker – selected from the TCGA analysis as one of the significantly different between PR+ and PR- patients, involved in signal transduction from membrane [7–9]
NM_001128431	SLC39A14 (Solute Carrier Family 39 Member 14)	PR downstream activation marker – selected from the TCGA analysis as one of the significantly different between PR+ and PR- patients
NM_001363568	UCK2 (Uridine-Cytidine Kinase 2)	PR downstream activation marker – selected from the TCGA analysis as one of the significantly different between PR+ and PR- patients [7–9]
NM_001020658	PUM1 (pumilio RNA binding family member 1)	House-keeping gene recommended by Nanostring
NM_004168	SDHA (succinate dehydrogenase complex flavoprotein subunit A)	House-keeping gene recommended by Nanostring
NM_003194	TBP (TATA-box binding protein)	House-keeping gene recommended by Nanostring

Table S4. Clinical and pathological characteristics of PR(mol-) versus PR(mol+) subgroups within ER+ patients. Nominal variables are presented as raw values followed by percentages of the respective groups, continuous variables are presented as medians and interquartile ranges in brackets.

**Variable**	**PR(mol-)** ***n*** **= 110 (31.8)**	**PR(mol+)** ***n*** **= 204 (59.0)**	***p*-value**
Age [years] [1]	65.0 (58.0–70.0)	63.0 (52.0–71.2)	0.125
Menopausal status [2]			
Pre	8 (8.2)	25 (13.3)	0.197
Post	90 (91.8)	163 (86.7)	
Grade [2]			
1	10 (9.1)	30 (14.7)	0.039 *
2	70 (63.6)	141 (69.1)	
3	30 (27.3)	33 (16.2)	
Ki67 [%] [1]	22.0 (10.0–40.0)	12.0 (5.0–22.0)	0.003 *
HER2-positivity [2]	19 (17.3)	13 (6.4)	0.002 *
Tumour size [mm] [1]	22.5 (17.0–30.0)	20.0 (15.0–25.0)	0.012 *
T feature [2]			0.007 *

pT1	29 (42.6)	99 (60.0)	
pT2	33 (48.5)	63 (38.2)	
pT3/4	6 (8.8)	3 (1.8)	
Metastases present	37 (34.6)	68 (34.0)	0.919
N feature [2]			
pN0	70 (65.4)	135 (67.5)	0.682
pN1	24 (22.4)	47 (23.5)	
pN2-3	13 (12.2)	18 (9.0)	
Staging [2]			
Very early (Stage IA)	36 (33.3)	86 (43.0)	0.220
Early (IB-IIIA)	64 (59.3)	104 (52.0)	
Advanced (IIIB-C, IV)	8 (7.4)	10 (5.0)	
Multifocality [2]	8 (11.8)	27 (16.4)	0.372
DCIS present [2]	35 (31.8)	56 (27.5)	0.416
DFS events [3]	14 (14.1)	21 (12.5)	0.354
Disease-free survival [years]	3.8 (2.3–4.9)	4.3 (2.8–6.7)	
Deaths [3]	11 (10.0)	20 (9.8)	0.551
Overall survival [years]	4.0 (2.8–5.5)	4.7 (3.0–6.7)	

[1] Mann-Whitney U test, [2] Pearson's chi-squared test, [3] log-rank test, * significant differences.

Table S5. Expression of FGFR2 protein and mRNA in PR(mol-) versus PR(mol+) patients within ER+ subgroup. Nominal variables are presented as raw values followed by percentages of the respective groups, continuous variables are presented as medians and interquartile ranges in brackets.

**Variable**	**PR(mol-)** ***n* = 110** **(35.0)**	**PR(mol+)** ***n* = 204** **(65.0)**	***p*-value**
FGFR2 protein [H-score] [1]	103.5 (21.0–210.0)	105 (22.0–201.5)	0.739
FGFR2 by H-score quartiles [2]			
0–75	44 (40.0)	77 (37.8)	0.929
76–150	22 (20.0)	47 (23.0)	
151–225	20 (18.2)	38 (18.6)	
226–300	24 (21.8)	42 (20.6)	
FGFR2 mRNA [log2] [1]	8.3 (7.2–9.3)	8.7 (8.1–9.4)	0.008 *

[1] Mann-Whitney U test, [2] Pearson's chi-squared test, * significant differences.

Table S6. Multivariate analyses of the combined effect of FGFR2 (protein) status and PR(mol) status on the poor prognostic associations characterised for FGFR2(low). The analysis involves only ER+ patients. Hazard ratios with confidence intervals are present for overall and disease-free survival, β-parameters and standard deviation for Ki67 proliferation index and odds ratios (OR) with confidence intervals for tumor grade.

**Feature**	**FGFR2 status (high as reference)**	**PR(mol) status (PR(mol+) as reference)**
Tumor grade (probability for grade 3 regarding grade 1)	OR 4.76 (95% CI 1.69–12.50); $p = 0.003$	OR 2.86 (95% CI 1.15–7.14); $p = 0.023$
Ki67 proliferation index	B = 5.1 (±1.77), p=0.005	β = 5.1 (±1.77), $p = 0.005$
Overall survival	HR 2.25 (95% CI 1.11–4.58), $p = 0.025$	HR 1.32 (95% CI 0.63–2.77), $p = 0.462$
Disease-free survival	HR 2.07 (95% CI 1.06–4.05), $p = 0.034$	HR 1.50 (95% CI 0.76–2.98), $p = 0.246$

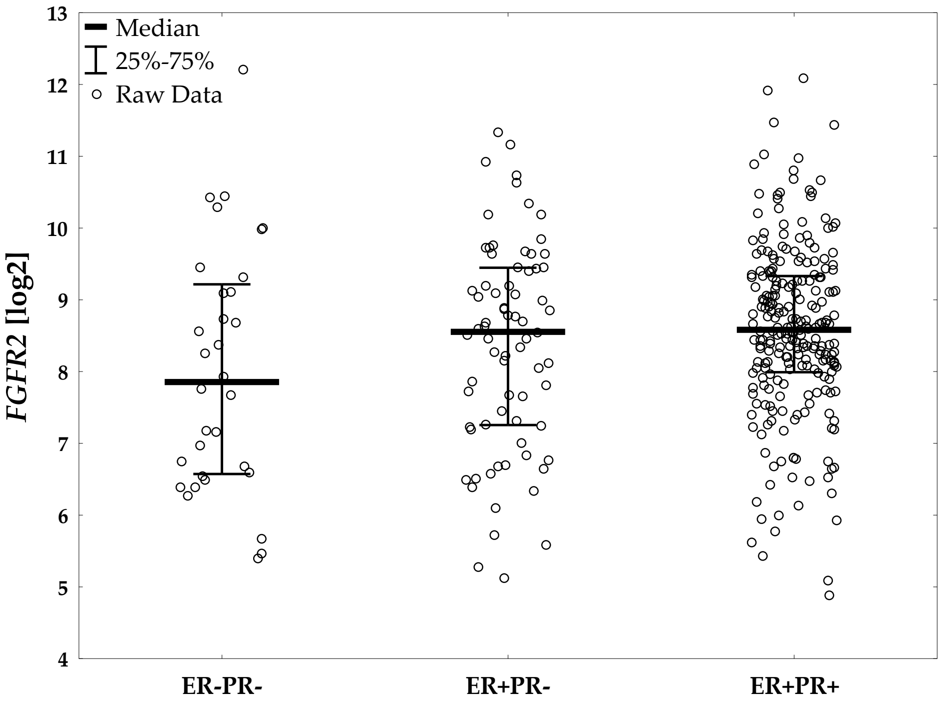

Figure S1. *FGFR2* mRNA levels compared between hormonal receptor status subgroups (ER-PR- vs. ER+PR- vs. ER+PR+), $p = 0.049$ from ANOVA test.

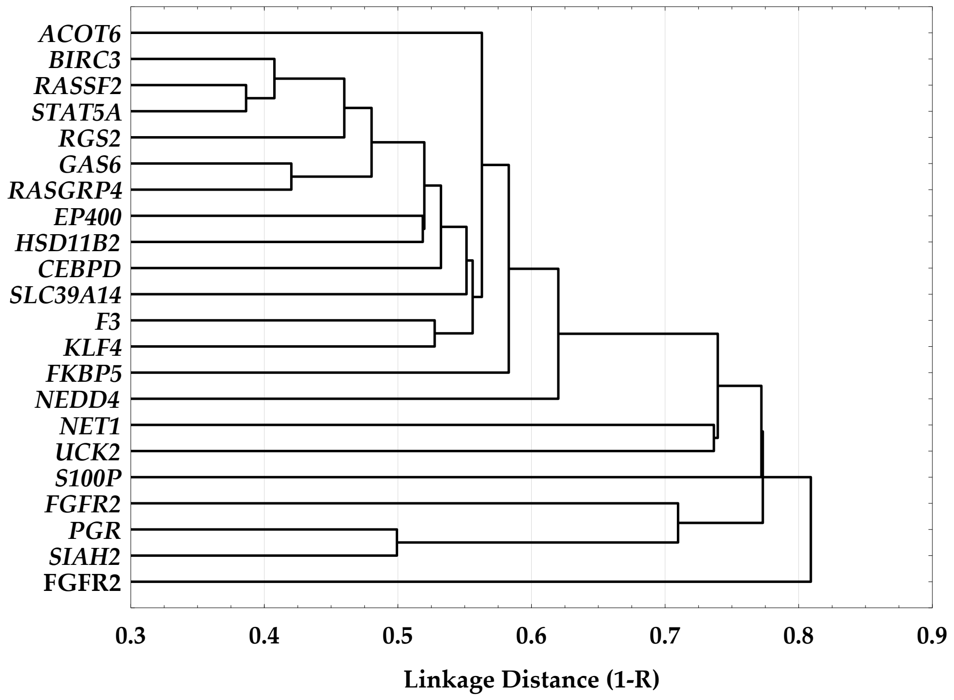

Figure S2. Hierarchical tree-clusterisation of genes included in PR-dependent molecular signature. All patients with good quality RNA were included in this analysis. Linkage distance is showed as 1-R (Spearman).

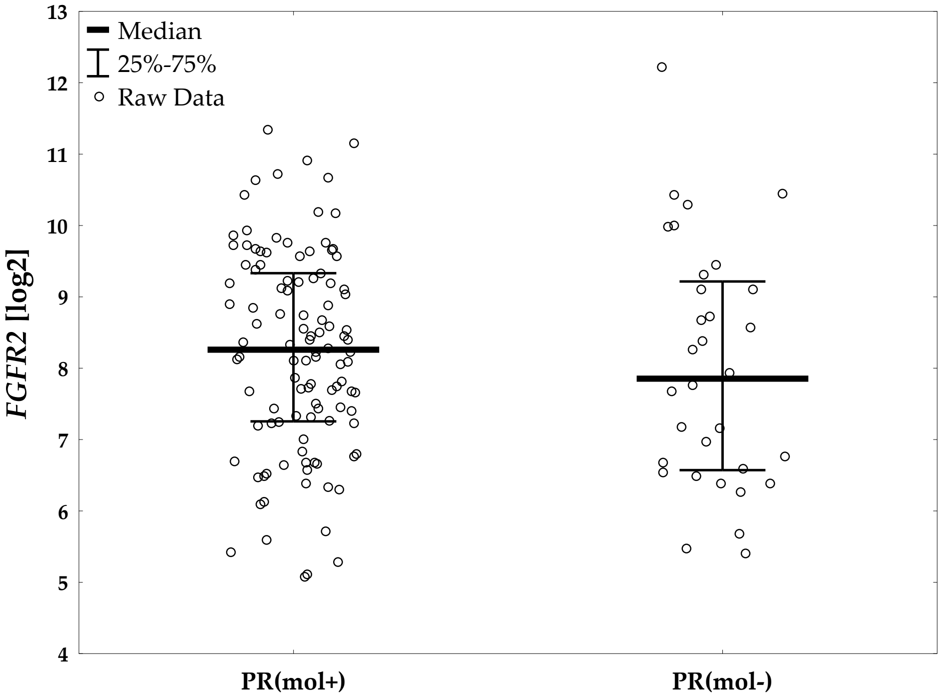

Figure S3. *FGFR2* mRNA levels compared between estrogen receptor status and progesterone receptor molecular activity status (ER-PR(mol-) vs. ER+PR(mol-) vs. ER+PR(mol+)), $p = 0.002$. *p*-value from ANOVA test.

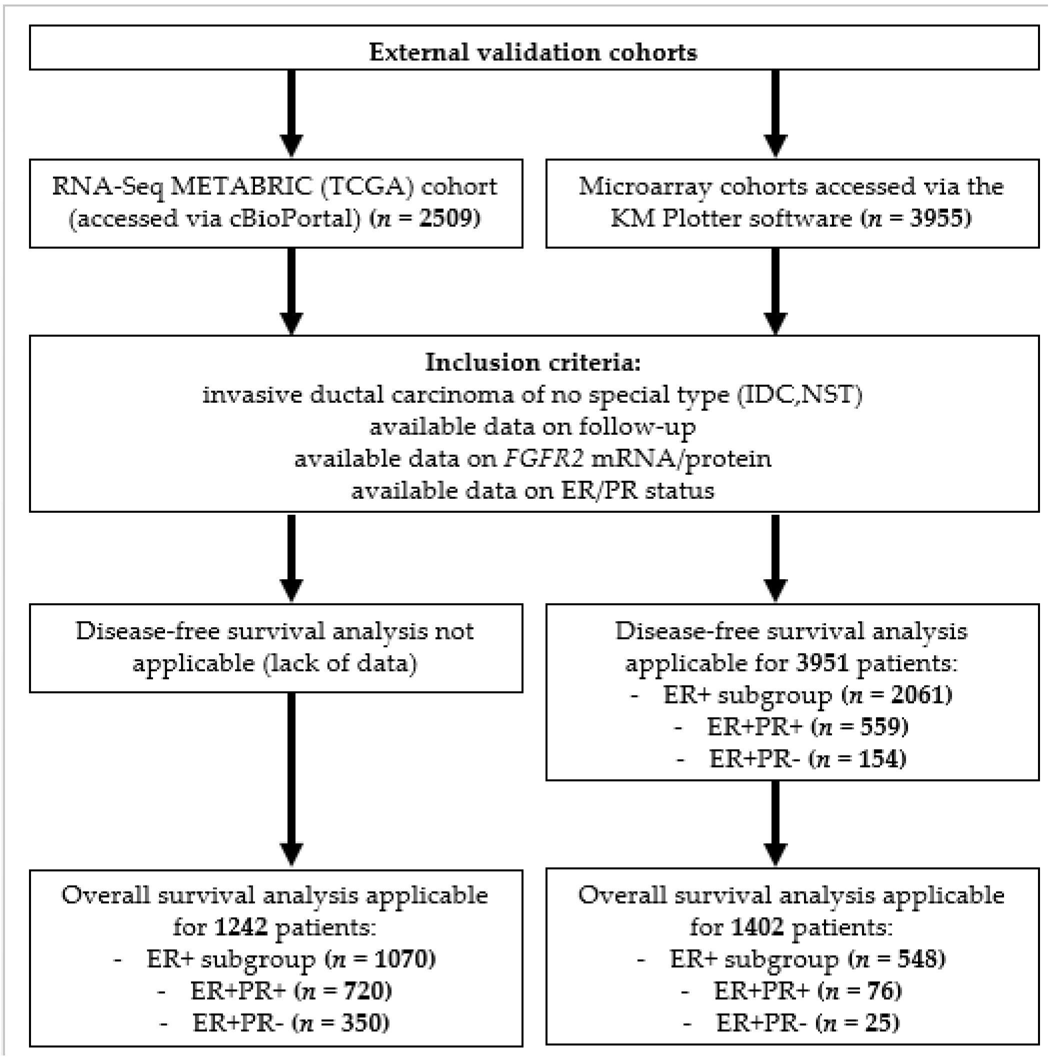

Figure S4. Flowchart of the in silico verification with indication of numbers of patients from the external databases included in every analysis. “ER+” subgroup included all ER+ patients regardless of PR status and it comprised ER+PR+ and ER+PR- subgroups. IDC, NST – invasive ductal carcinoma of no special type, ER – estrogen receptor protein status, PR – progesterone receptor protein status, FGFR2 – fibroblast growth factor receptor 2 protein.

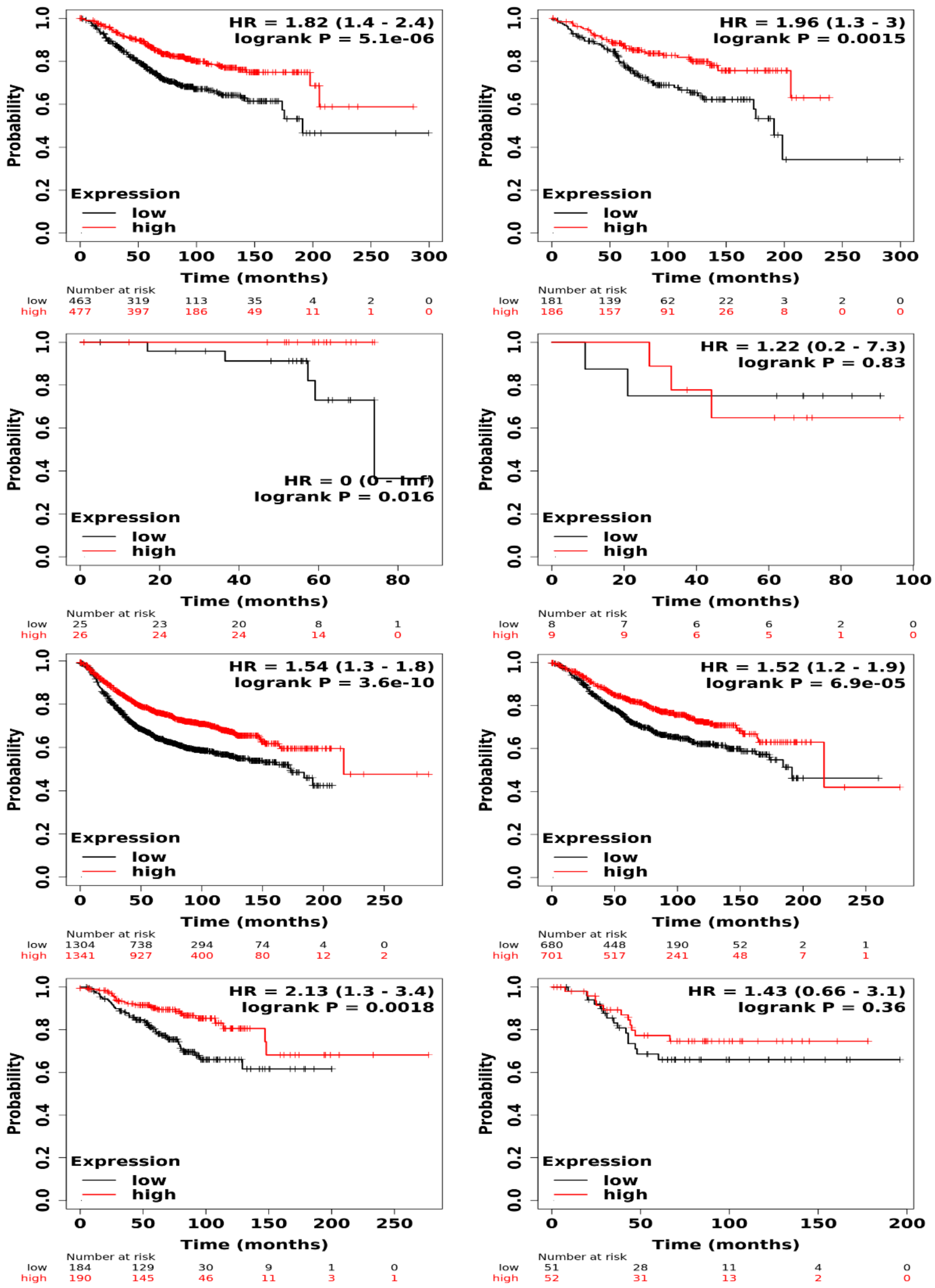

Figure S5. Kaplan-Meier curves for OS (**a**–**d**) and PFS (**e**–**h**) regarding FGFR2 microarray mRNA levels. "ER+" subgroup included all ER+ patients regardless of PR status and it comprised ER+PR+ and ER+PR- subgroups. FGFR2low stands for 1st tercile and FGFR2high for 2nd-3rd terciles. Plots were generated using online open access tool KM plotter (encompassing patients different than those included in TCGA database). (**a**) OS probability in all 1402 breast cancer patients, (**b**) OS probability in all 548 ER+ breast cancer patients, (**c**) OS probability in all 76 ER+PR+ patients, (**d**) OS probability in all 25 ER+PR- patients., (**e**) PFS probability in all 3951 breast cancer patients, (**f**) PFS probability in all 2061 ER+ breast cancer patients, (**g**) PFS probability in all 559 ER+PR+ patients, (**h**) PFS probability in all 154 ER+PR- patients.

References

1. Sinn, H.-P.; Schneeweiss, A.; Keller, M.; Schlombs, K.; Laible, M.; Seitz, J.; Lakis, S.; Veltrup, E.; Altevogt, P.; Eidt, S.; et al. Comparison of immunohistochemistry with PCR for assessment of ER, PR, and Ki-67 and prediction of pathological complete response in breast cancer. *BMC Cancer* **2017**, *17*, 1–10, doi:10.1186/s12885-017-3111-1.
2. Laible, M.; Schlombs, K.; Kaiser, K.; Veltrup, E.; Herlein, S.; Lakis, S.; Stöhr, R.; Eidt, S.; Hartmann, A.; Wirtz, R.M.; et al. Technical validation of an RT-qPCR in vitro diagnostic test system for the determination of breast cancer molecular subtypes by quantification of ERBB2, ESR1, PGR and MKI67 mRNA levels from formalin-fixed paraffin-embedded breast tumor specimens. *BMC Cancer* **2016**, *16*, 1–14, doi:10.1186/s12885-016-2476-x.
3. Varga, Z.; Lebeau, A.; Bu, H.; Hartmann, A.; Penault-Llorca, F.; Guerini-Rocco, E.; Schraml, P.; Symmans, F.; Stoehr, R.; Teng, X.; et al. An international reproducibility study validating quantitative determination of ERBB2, ESR1, PGR, and MKI67 mRNA in breast cancer using MammaTyper®. *Breast Cancer Res.* **2017**, *19*, 55, doi:10.1186/s13058-017-0848-z.
4. Wilson, T.R.; Xiao, Y.; Spoerke, J.M.; Fridlyand, J.; Koeppen, H.; Fuentes, E.; Huw, L.Y.; Abbas, I.; Gower, A.; Schleifman, E.B.; et al. Development of a robust RNA-based classifier to accurately determine ER, PR, and HER2 status in breast cancer clinical samples. *Breast Cancer Res. Treat.* **2014**, *148*, 315–325, doi:10.1007/s10549-014-3163-8.
5. Jansen, M.P.; Ruigrok-Ritstier, K.; Dorssers, L.C.; van Staveren, I.L.; Look, M.P.; Meijer-van Gelder, M.E.; Sieuwerts, A.M.; Helleman, J.; Sleijfer, S.; Klijn, J.G.; et al. Downregulation of SIAH2, an ubiquitin E3 ligase, is associated with resistance to endocrine therapy in breast cancer. *Breast Cancer Res. Treat.* **2009**, *116*, 263–271, doi:10.1007/s10549-008-0125-z.
6. Santos, S.J.; Aupperlee, M.D.; Xie, J.; Durairaj, S.; Miksicek, R.; Conrad, S.E.; Leipprandt, J.R.; Tan, Y.S.; Schwartz, R.C.; Haslam, S.Z. Progesterone receptor A-regulated gene expression in mammary organoid cultures. *J. Steroid Biochem. Mol. Biol.* **2009**, *115*, 161–172, doi:10.1016/j.jsbmb.2009.04.001.
7. Richer, J.K.; Jacobsen, B.M.; Manning, N.G.; Abel, M.G.; Wolf, D.M.; Horwitz, K.B. Differential gene regulation by the two progesterone receptor isoforms in human breast cancer cells. *J. Biol. Chem.* **2002**, *277*, 5209–5218, doi:10.1074/jbc.M110090200.
8. Knutson, T.P.; Daniel, A.R.; Fan, D.; Silverstein, K.A.; Covington, K.R.; Fuqua, S.A.; Lange, C.A. Phosphorylated and sumoylation-deficient progesterone receptors drive proliferative gene signatures during breast cancer progression. *Breast Cancer Res.* **2012**, *14*, 1–23, doi:10.1186/bcr3211.
9. Daniel, A.R.; Knutson, T.P.; Lange, C.A. Signaling inputs to progesterone receptor gene regulation and promoter selectivity. *Mol. Cell Endocrinol.* **2009**, *308*, 47–52, doi:10.1016/j.mce.2009.01.004.
